# A Patient-Centered Understanding of the Referral System in Ethiopian Primary Health Care Units

**DOI:** 10.1371/journal.pone.0139024

**Published:** 2015-10-05

**Authors:** Orit Abrahim, Erika Linnander, Halima Mohammed, Netsanet Fetene, Elizabeth Bradley

**Affiliations:** Yale University School of Public Health, New Haven, Connecticut, United States of America; University College London, UNITED KINGDOM

## Abstract

**Background:**

Primary healthcare systems in sub-Saharan Africa have undergone substantial development in an effort to expand access to appropriate facilities through a well-functioning referral system. The objective of this study was to evaluate the current patterns of seeking prior care before arriving at a health center or a hospital as a key aspect of the referral system of the primary health care unit (PHCU) in three regions in Ethiopia. We examined what percentage of patients had either sought prior care or had been referred to the present facility and identified demographic and clinical factors associated with having sought prior care or having been referred.

**Methods and Findings:**

We conducted a cross-sectional study using face-to-face interviews in the local language with 796 people (99% response rate) seeking outpatient care in three primary health care units serving approximately 100,000 people each and reflecting regional and ethnic diversity; 53% (N = 418) of the sample was seeking care at hospital outpatient departments, and 47% of the sample was seeking care at health centers (N = 378). We used unadjusted and adjusted logistic regression to identify factors associated with having been referred or sought prior care. Our findings indicated that only 10% of all patients interviewed had been referred to their current place of care. Among those in the hospital population, 14% had been referred; among those in the health center population, only 6% had been referred. Of those who had been referred to the hospital, most (74%) had been referred by a health center. Among those who were referred to the health center, the plurality portion (32%) came from a nearby hospital (most commonly for continued HIV treatment or early childhood vaccinations); only 18% had come from a health post. Among patients who had not been formally referred, an additional 25% in the hospital sample and 10% in the health center sample had accessed some prior source of care for their present health concern. In the adjusted analysis, living a longer distance from the source of care and needing more specialized care were correlated with having sought prior care in the hospital sample. We found no factors significantly associated with having sought prior care in the health center sample.

**Conclusions:**

The referral system among health facilities in Ethiopia is used by a minority of patients, suggesting that intended connections between health posts, health centers, and hospitals may need strengthening to increase the efficiency of primary care nationally.

## Introduction

Strengthening primary health care is essential to improving access and quality of health care in low-income countries [1]. Nevertheless, implementing effective interventions to strengthen primary care has been challenging, and many efforts have had limited success because of insufficient financial resources, weak political engagement, or inadequate management of the referral patterns of patients using primary health centers and secondary hospitals. A key element of primary health care is its referral system in which patients are able to access care at community-based health posts or health centers before accessing higher-levels of care such as secondary and tertiary hospitals. Some communities may depend on and prefer indigenous healers, customs, and knowledgeable family members; however, effective linkages between health posts and health centers and between health centers and hospitals may nonetheless enhance access to services not attained through alternative means.

Despite the importance of understanding the linkages among health facilities within a primary health care system, we lack contemporary studies that have reported such data in low-income settings. We could find only five relevant studies from low-income countries [2–5]. Studies from Zimbabwe [2], Nigeria [3], and Namibia [4] each reported that a minority of people (38%, 7%% and between 27%-52% depending on the facility, respectively) seeking care at hospitals had been referred from a more primary source of care, with the majority accessing the hospital as their first source of care. Furthermore, in the study from Zimbabwe [2], researchers suggested that more than half the patients who were referred from a health center to a hospital should have been treated at lower levels of care. The study from Niger [6] identifies reasons for the study facility to issue a referral but does not quantify the percentage of patients who had sought more primary care before arriving at the study facility and whether or not they were formally referred. Although these studies are helpful, their evidence is limited, and all but one [6] are more than a decade old. Thus, despite substantial efforts to strengthen primary care, the degree to which primary care referral systems are functioning efficiently in sub-Saharan Africa remains largely unknown.

Accordingly, we sought to quantify the percentage of patients who accessed other facilities in the primary health care system before presenting to hospitals and health centers, and to identify the clinical and demographic factors associated with having accessed prior care. We examined both those that had a formal referral and those who may not have been formally referred but did visit a more primary source of care prior to seeking care at the hospital or health center. We chose to conduct this study in Ethiopia as an ideal setting given their enormous investment in the Ethiopian Health Extension Program since 2003 [7]. Our findings will provide contemporary data on referral patterns and may be helpful in targeting interventions to promote more efficient use of primary health care services in low-income countries with developing PHCUs.

## Methods

### Ethics Statement

All consent and research procedures were approved by the Human Research Protection Program, Human Subjects Committee (HSC) at the Yale School of Medicine and the Ethiopian Ministry of Health. We obtained exemption (protocol number 1405013942) which waived the need for participant consent because no identifying personal health information was obtained. All participants were provide with an information sheet to inform them about the objective of the research, let them know what data would be collected, how it would be used and disseminated, and any risks that would be encountered by participation. Oral consent was most appropriate considering the high rate of illiteracy in rural study sites and the desire to capture a large representative sample. After being informed about the study, risks and benefits, all possible participants were asked if they would be willing to complete a short verbally-administered survey. Because we obtained verbal consent, documentation of consent was not required. The response rate for each sample was 99% with 1% preferring not to participate.

### Setting

Since 2006, Ethiopia has invested heavily in its primary care system with the goal of providing accessible and high quality care to its population, 85% of which live rurally [8]. The Primary Health Care Unit (PHCU) has been the cornerstone of the primary care system and consists of care for a population of about 100,000 people including on average 4 health centers, 20 health posts, 40 health extension workers (HEWs), and, recently, a primary hospital. Across the country as of 2014 the Health Extension Program of the Federal Ministry of Health has trained and deployed more than 35,000 HEWs and created over 8,000 health posts [9]. Rapid expansion and reorganization of primary health care infrastructure is apparent; however, the degree to which the PHCU framework [10] has changed referral patterns is largely unknown.

### Study Design and Sample

We conducted a cross-sectional study of 3 PHCUs that were purposefully selected to reflect regional and ethnic diversity. In each PHCU (located in Oromia, Tigray, and Southern Nation’s Nationalities’ and People’s Region), we selected 1 hospital and 1–2 health centers with the highest volume during the study period (June-July 2014) in which we conducted consecutive face-face interviews with all people who arrived at the medical records office seeking outpatient or emergency care during the working day for the 5-day study period in each PHCU. For patients who were younger than 18 years old or physically or mentally unable to complete the survey, a parent or relative completed the survey as a proxy (this occurred in 26.4% of cases). Interviews were conducted at the health center and hospital in the local language until similar sample sizes were reached for each facility type (N = 418 hospital and N = 378 health center for a total sample of N = 796 total). Sample sizes from each PHCU reflect their respective facility volumes and catchment areas (Oromia: N = 354, Tigray: N = 287, Southern Nation’s Nationalities’ and People’s Region: N = 155).

### Data Collection

**Fig 1:**
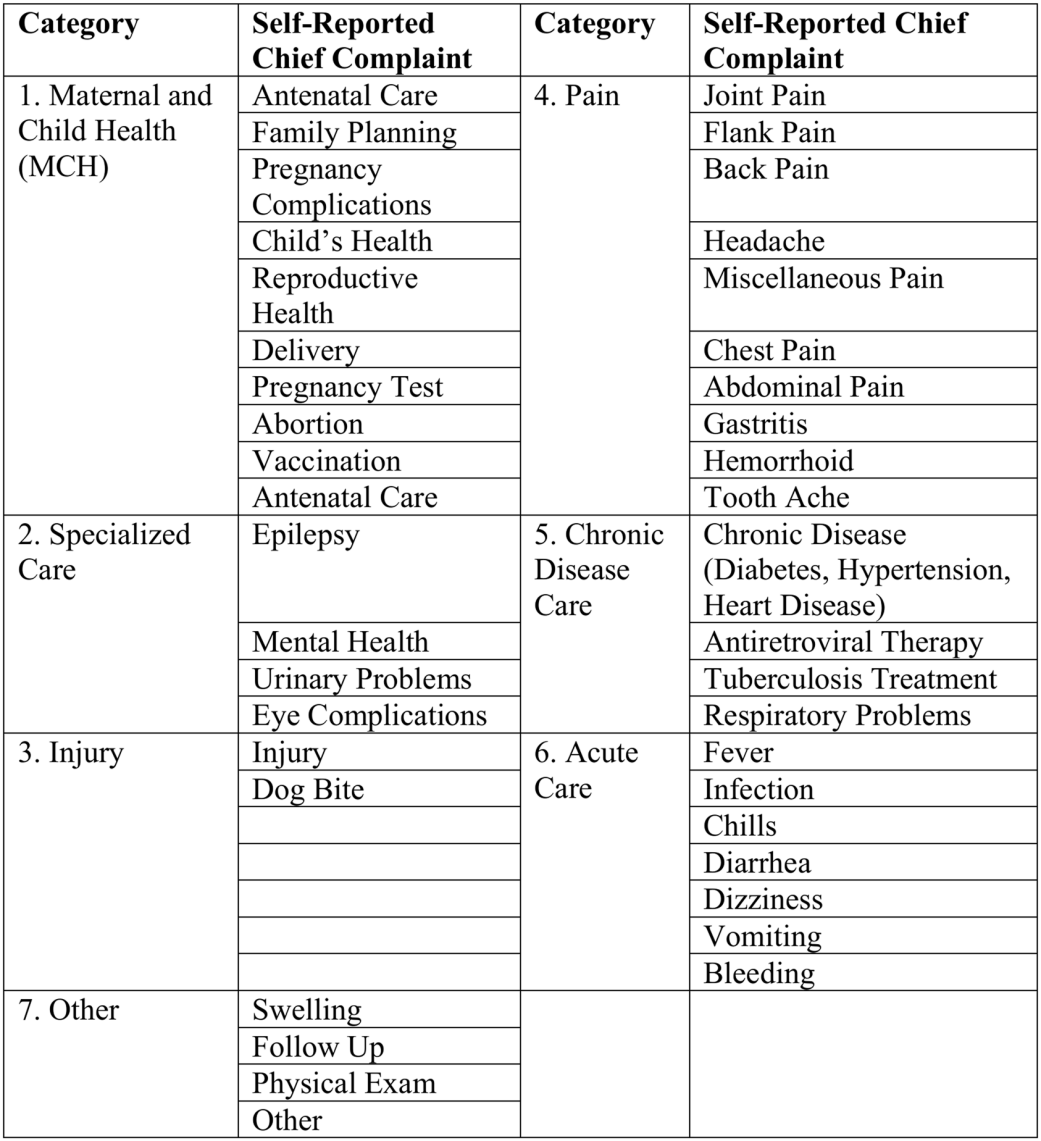
Categorization of Chief Complaint.

The primary outcomes were: 1) whether the patient was referred to the present facility by another health care provider or organization (public or private sector), and 2) whether the patient sought prior care (regardless of if he or she had a formal referral). Independent variables included patient demographic factors (gender, age, and distance between residence and source of care), and chief complaint. Chief complaint was classified in 1 of 7 categories: maternal and child health, pain, injury, specialized care, chronic care, acute care, and other. Criteria for inclusion in the group were based on similarity of symptoms and treatment (Fig 1). If multiple chief complaints were given, the complaint was classified according to the first compliant recorded (typically the primary complaint).

### Data Analysis

We used standard frequency analysis to describe the sample and prevalence of outcomes variables. We compared the demographic and clinical characteristics of people accessing care at health centers and hospitals using chi-square statistics for categorical variables and t-tests for continuous variables. We used separate multivariable logistic regression models to identify factors associated with being referred and seeking prior care, and we reported unadjusted and adjusted odds ratios and 95% confidence intervals. We fit regression models using backward regression retaining significant variables and variables whose removal resulted in changes in remaining parameter estimates of more than 20% and thus deemed confounders. All data analyses were performed using Excel and Statistical Analysis System (SAS), version 9.3.

## Results

The majority of patients from health centers (N = 378) and hospitals (N = 418) were female (71.4%) with a mean age of 31 years (Table 1). People accessing a health center compared with those accessing a hospital were more likely to be female (82.2% and 61.2%, respectively, P-value < 0.001). Almost half of all respondents sought maternal and child health care (MCH), and this was more common among the patients accessing the health center than for those accessing the hospital (59.7% versus 40.6%, respectively, P-value < 0.001). Pain and specialized care needs were more prevalent among patients at the hospital compared with health center. Approximately 76% of patients lived within 1–10 km of the health facility, and people seeking care at the hospital compared with the health center were more likely to have traveled farther than 20 km to access care (27.5% and 5.3%, respectively, P-value < 0.001).

**Table 1:** Descriptive statistics by type of facility.

	Total Sample	Hospital Sample	Health center sample	P-value^3^
	(N = 796) N (%)	(N = 418) N (%)	(N = 378) N (%)	
**Gender**				< 0.001
Female	550 (71.4)	241 (61.2)	309 (82.2)	
Male	220 (28.6)	153 (38.8)	67 (17.8)	
Missing	26	24	2	
**Mean age** ^**1**^ **(Standard deviation)**	31.0 (12.6)	33.4 (14.6)	28.8 (9.7)	< 0.001
**Distance between residence and source of care** ^**2**^				< 0.001
1–10 km	478 (75.6)	228 (65.3)	250 (88.3)	
11–20 km	43 (6.8)	25 (7.2)	18 (6.4)	
> 20km	111 (17.6)	96 (27.5)	15 (5.3)	
**Chief Complaint**				< 0.001
Pain	123 (15.5)	86 (20.7)	37 (9.8)	
Maternal and Child Health	394 (49.7)	169 (40.6)	225 (59.7)	
Injury	36 (4.5)	20 (4.8)	16 (4.2)	
Specialized Care	37 (4.7)	32 (7.7)	5 (1.3)	
Chronic Care	99 (12.5)	50 (12.0)	49 (13.0)	
Acute Care	26 (3.3)	14 (3.4)	12 (3.2)	
Other or missing	81 (10.1)	47 (11.2)	34 (9.0)	
**Prior Care**				<0.001
Yes	221 (28.0)	161 (38.7)	60 (16.1)	
No	568 (72.0)	255 (61.3)	313 (83.9)	
Missing	7	2	5	
**Referred**				<0.001
Yes	79 (9.9)	57 (13.6)	22 (5.8)	
No	717 (90.1)	361 (86.4)	356 (94.2)	

^1^ 43 people did not report their age.

^2^ 164 people did not know the distance

^3^ P-Values calculated using chi-squared test for categorical variables and Student’s t-test for continuous variable age.

### Proportion referred from or having received prior source of care

A total of 9.9% of the overall sample had been referred from a previous health care provider. Among the hospital sample, 13.6% of patients had been referred from another source of care; among the health center sample, only 5.8% of patients had been referred from another source of care. Although not formally referred, an additional 25.1% of the hospital sample and an additional 10.3% of the health center sample had sought prior care.

### Previous sources of care

Of the 79 (9.9%) people in both samples who had been referred from a previous source of care, 42 people (53.2%) had been referred from a health center to a hospital, and only 4 people (5.1%) had been referred from a health post or health extension worker to a health center (Table 2). Of those who were referred to the hospital, most came from the health center (73.7%), whereas among those who were referred to the health center, most came from a primary hospital (31.8%) as back referrals for continued HIV treatment or early childhood vaccinations. Of the added 104 people who were not formally referred but had received prior care before arriving to the hospital, the most common place for the prior care was a health center (for 53.7% of the people who were not formally referred but who had received prior care). Of the added people who were not formally referred but had sought prior care before arriving at a health center, the most common places of prior care were a health center (28.6%), a primary hospital (27.0%), or a private clinic (23.8%).

**Table 2:** Sources of prior care and sources of referrals.

	Hospital sample		Health center sample	
Source of prior care	People who sought prior care by source of care	People who were referred by source of referral	People who sought prior care by source of care	People who were referred by source of referral
	N (%)	N (%)	N (%)	N (%)
Health center	94 (53.7)	42 (73.7)	18 (28.6)	4 (18.2)
Health post/HEW^1^	2 (1.1)	2 (3.5)	6 (9.5)	4 (18.2)
Hospital in PHCU^2^	28 (16.0)	2 (3.5)	17 (27.0)	7 (31.8)
Private clinic	23 (13.1)	3 (5.3)	15 (23.8)	5 (22.7)
Hospital in Addis	7 (4.0)	4 (50.0)	3 (4.8)	1 (4.5)
Pharmacy	6 (3.4)	0 (0.0)	2 (3.2)	0 (0.0)
University Clinic	4 (2.3)	1 (12.5)	0 (0.0)	0 (0.0)
District Hospital	6 (3.4)	0 (0.0)	0 (0.0)	0 (0.0)
Other^3^	5 (2.9)	3 (37.5)	2 (3.2)	1 (4.5)
N^4^	175	57	63	22

^1^ HEW: Health extension worker

^2^ PHCU: Primary health care unit

^3^ Other includes follow up, non-governmental organizations, Emmanuel Mental Health hospital, care abroad, and traditional medicine.

^4^ N = 13 people seeking care at a hospital had sought care at more than 1 prior facility, and N = 3 people seeking care at a health center had sought care at more than 1 prior facility.

### Factors associated with having been referred

For the hospital sample, living more than 20 kilometers compared with 10 or less kilometers from the hospital was associated with having been referred in both unadjusted and adjusted analyses (P-values < 0.05) (Table 3). In unadjusted analysis, having the chief complaint of injury (with reference group being maternal and child health needs) was associated with having been referred but this was non-significant in adjusted analysis. For the health center sample, having the chief complaint of chronic care—with a majority seeking HIV treatment—was associated with having been referred, although this was non-significant in adjusted analysis.

**Table 3:** Factors associated with being referred from prior source of care.

	Hospital sample (N = 418) Odds ratio (OR) of having been referred		Health center sample (N = 378) OR of having been referred	
	Unadjusted OR (95% CI)	Adjusted OR (95%CI)	Unadjusted OR (95% CI)	Adjusted OR (95% CI)
**Gender**				
Female	*Reference*	*Reference*	*Reference*	*Reference*
Male	1.49 (0.83–2.66)	0.75 (0.30–1.86)	0.72 (0.21–2.49)	0.40 (0.02–8.80)
**Distance between residence and care**				
1–10 km	*Reference*	*Reference*	*Reference*	Non-significant
11–20 km	0.96 (0.21–4.37)	1.32 (0.25–6.91)	3.37 (0.87–13.03)	
> 20km	3.87 (2.01–7.45)*	3.07 (1.21–7.74)*	1.20 (0.15–9.83)	
**Chief complaint**				
Maternal and Child Health	*Reference*	*Reference*	*Reference*	*Reference*
Pain	0.92 (0.40–2.14)	0.40 (0.11–1.48)	0.54 (0.07–4.31)	0.27 (0.02–4.87)
Injury	4.25 (1.51–11.97)*	3.61 (0.76–17.08)	N/A	Non-significant
Specialized Care	2.21 (0.84–5.80)	1.04 (0.22–4.87)	4.86 (0.50–47.24)	Non-significant
Chronic Care	1.50 (0.62–3.68)	0.80 (0.18–3.45)	3.24 (1.19–8.85)*	0.96 (0.03–27.98)
Acute Care	0.61 (0.08–4.91)	0.75 (0.08–7.15)	N/A	Non-significant
Other	1.22 (0.45–3.25)	0.81 (0.14–4.76)	1.26 (0.27–5.93)	Non-significant

CI: Confidence interval

*P-Value < 0.05

### Factors associated with having sought prior care

For the hospital sample, living more than 20 kilometers compared with 10 or less kilometers from the hospital was associated with having sought prior care in both unadjusted and adjusted analyses (P-values < 0.05) (Table 4). Chief complaint was also significant in that people with pain or chronic disease were more likely to have sought prior care in unadjusted analysis, and in adjusted analysis those needing specialized care were less likely to have sought prior care. For the health center sample, living more than 20 kilometers compared with 10 or less kilometers from the health center was associated with having sought a prior source of care.

**Table 4:** Factors Associated with Seeking Prior Care of Patients at Hospital and Health Center.

	Hospital sample (N = 418) Odd ratio (OR) of seeking prior care		Health Center sample (N = 378) OR of seeking prior care	
	Unadjusted OR (95% CI)	Adjusted OR (95%CI)	Unadjusted OR (95% CI)	Adjusted OR (95%CI)
**Gender**				
Female	*Reference*	*Reference*	*Reference*	*Reference*
Male	1.19 (0.78–1.81)	0.79 (0.44–1.43)	1.32 (0.67–2.61)	1.32 (0.37–4.67)
**Distance between residence and care**				
1–10 km	*Reference*	*Reference*	*Reference*	*Reference*
11–20 km	1.77 (0.77–4.10)	1.714 (0.61–4.86)	1.49 (0.47–4.77)	Not applicable
> 20km	3.30 (2.01–5.41)*	2.66 (1.40–5.04)*	2.61 (0.85–8.05)	6.45 (1.01–41.33)*
**Chief complaint**				
Maternal and Child Health	*Reference*	*Reference*	*Reference*	*Reference*
Pain	1.90 (1.12–3.25)*	0.89 (0.41–1.93)	1.08 (0.39–3.01)	0.75 (0.14–4.17)
Injury	1.79 (0.70–4.58)	0.96 (0.26–3.56)	0.46 (0.06–3.63)	0.64 (0.06–7.36)
Specialized Care	1.15 (0.52–2.55)	0.30 (0.09–0.97)*	4.62 (0.74–28.89)	Non-significant
Chronic Care	2.79 (1.46–5.31)*	1.46 (0.54–3.95)	2.57 (1.22–5.45)*	3.03 (0.42–22.00)
Acute Care	0.88 (0.26–2.92)	0.68 (0.17–2.82)	3.46 (0.98–12.26)	1.71 (0.22–13.23)
Other	1.25 (0.62–2.51)	0.71 (0.24–2.10)	1.87 (0.74–4.70)	0.35 (0.03–4.92)

CI: Confidence interval

*P-Value < 0.05

## Discussion

Despite the remarkable expansion in the number of health posts and health centers in Ethiopia, we found that people routinely accessed hospitals without a formal referral from a health center or health post and without seeking any prior source of care. Only a small minority of patients who accessed health centers had first been seen at a health post or by a health extension worker. Although people seeking care for illness are not expected to visit a health post before a health center, the government of Ethiopia invested in developing the primary health care system to enable people to more easily seek care prior to hospital care. Nevertheless, the substantial majority of patients access hospitals directly, without referral and without seeking prior sources of care.

Our finding indicates that, although the primary health care elements (e.g., health center, health posts, and health extension workers) may be providing essential care and prevention resources for rural populations in Ethiopia, people often access secondary levels of care without first using primary health care provided by health centers and health posts. Our clinical data were not detailed enough to assess the clinical appropriateness of the observed patterns of care; however, our results show that about 40% of people who arrived at the hospital were seeking routine maternal and child health care, which included services such as antenatal care, family planning, and childhood vaccinations that are included in standard practices of health centers and health posts in Ethiopia. At least in these cases, it appears that services available at health posts and health centers were bypassed to access hospital-based care, a pattern similar to that documented in older studies [2–5] from other sub-Saharan African countries.

The underlying reasons for this lack of prior care seeking cannot be known with our available data; however, several explanations are possible. First, some community members may prefer to consult with traditional healers or family members rather than the more formal primary care providers, and to proceed directly to the hospital when they believe the condition exceeds the capabilities of the local provider. A recent study that evaluated where pregnant women in rural Ethiopia decided to deliver suggested that traditional practices were not contradictory to more westernized services [11]. Rather, HEWs and traditional birth attendants were key in encouraging women to visit health facilities for antenatal care and delivery. Second, people may bypass health centers due to concerns about financial and non-financial costs, although much of the primary care in Ethiopia is free. Some studies have suggested that health centers may levy fees [12] and the time burden of visiting a health center may limit use of primary levels of care [13]. Last, people may perceive that health posts and health centers lack key services and medications desired by patients. In a 2013 study [13] on primary health care services in Ethiopia, providers described shortages in needed medications and supplies, as well as limitations in HEW training resulting in difficulties meeting population needs. This lack of comprehensiveness and quality—either perceived or real—may result in undue burden and crowding in hospitals.

While several reports have identified significant improvements in antenatal care coverage, skilled birth attendance rates, and HIV testing [14] as a result of primary health care reforms, and Ethiopia is on track to meet nearly all health Millennium Development Goals [15], addressing this pattern of bypassing primary health care services is paramount to sustaining the path of economic development and health improvement Ethiopia has begun. It is possible that with more efforts to educate rural denizens about the availability of health posts and health centers, the bypass pattern may attenuate, particularly if the services provided by these primary care providers meets people’s expectations and needs. Although it is important to recognize that some people may prefer less formal, traditional approaches to health care, several studies [11] [14] [16] have shown that, even in communities that rely on indigenous medicine, utilization of primary health care facilities can be increased through health education by community health workers and integration of cultural practices with westernized medicine. These studies also have found that such increased use of primary care facilities is a cost effective investment and can improve health outcomes.

Our findings should be interpreted in light of several limitations, although it is the first of its kind in Ethiopia and contributes importantly to contemporary literature on referral patterns in primary health care in low-income countries. Our sample, however, was derived from a small number of health facilities. Although we examined a diversity of regions and ethnic backgrounds, results may differ in other areas of Ethiopia we were unable to study. Second, due to limited electronic data collection tools in Ethiopia, data were collected on paper and transported to Addis Ababa before data entry, which may have resulted in errors. We tried to minimize errors in data collection and entry by repeated checks for consistency between records and the retention of original hard copy documents for reference. Last, we had no measure of clinical appropriateness in this study; however, examining the chief complaints suggested that at least some cases would likely be appropriate for primary, non-hospital care although more clinical research on this phenomenon is warranted.

In summary, this study describes common health care-seeking patterns of people living in rural Ethiopia and suggests one that is heavily concentrated in maternal and child health care, high rates of health center bypass to access hospitals directly, and little utilization of health posts for care prior to accessing health centers. Future reforms targeted at the primary health care unit should prioritize the appropriate distribution of quality maternal and child health care services at the health posts and health centers as these data demonstrated high demand for these services at both hospitals and health centers. Further research may be useful to assess the community’s understanding and acceptance of the role of each component of the primary health care unit in the decision to seek care, and the degree to which primary care facilities are limiting the overuse of more expensive secondary care.

## References

[1] Van Lerberghe W. The world health report 2008: primary health care: now more than ever. World Health Organization. 2008.

[2] Sanders D, Kravitz J, Lewin S, McKee M. Zimbabwe's hospital referral system: does it work? Health Policy and Planning. 1998;4:359–370. 10.1093/heapol/13.4.359 10346028

[3] Low A, Coeyere DD, Shivute N, Brandt LJ. Patient referral patterns in Namibia: identification of potential to improve the efficiency of the health care system. The International Journal of health planning and management. 2001;3:243–257. 10.1002/hpm.628 11596560

[4] Akande TM. Referral system in Nigeria: study of a tertiary health facility. Annals of African Medicine. 2004;3:130–133.

[5] Nordberg E, Holmberg S, Kiugu S. Exploring the interface between first and second level of care: referrals in rural Africa. Tropical Medicine & International Health.1996;1:107–111. 8673814 10.1046/j.1365-3156.1996.d01-2.x

[6] Bossyns P, Abache F, Abdoulaye MS, Miyé H, Depoorter AM, Van Lerberghe W. Monitoring the referral system through benchmarking in rural Niger: an evaluation of the functional relation between health centres and the district hospital. BMC health services research. 2006;1: 51. 10.1186/1472-6963-6-51 PMC1458337 16608534

[7] Federal Democratic Republic of Ethiopia MOH. 2010 Health Sector Development Program IV 2010/11-2014/15. 2010.

[8] Ringheim K, Teller C, Sines E. Ethiopia at a crossroads: Demography, gender, and development. Population Reference Bureau. 2009. Available: www.prb.org/policybrief.

[9] Federal Democratic Republic of Ethiopia MOH. 2010 Health Sector Development Program IV 2010/11-2014/15. 2010.

[10] Bekele A, Kefale M, Tadesse M. Preliminary assessment of the implementation of the health services extension program: the case of southern Ethiopia. Ethiop J Health Dev. 2008;22(3):302–305.

[11] Gebrehiwot T, Goicolea I, Edin K, Sebastian MS. Making pragmatic choices: women’s experiences of delivery care in Northern Ethiopia. BMC pregnancy and childbirth. 2012; 12(1), 113. 23078068 10.1186/1471-2393-12-113 PMC3542090

[12] Pearson L, Gandhi M, Admasu K, Keyes EB. User fees and maternity services in Ethiopia. International Journal of Gynecology & Obstetrics, 2011; 115(3), 310–315. 21982855 10.1016/j.ijgo.2011.09.007

[13] Sipsma H, Thompson J, Maurer L, Bradley E, Curry L. Preferences for home delivery in Ethiopia: provider perspectives. Global public health. 2013; 8(9):1014–1026. doi: 10.1080/17441692.2013.835434 24156727 10.1080/17441692.2013.835434

[14] Bradley E, Thompson JW, Byam P, Webster TR, Zerihun A, Alpern R, et al Access and quality of rural healthcare: Ethiopian Millennium Rural Initiative. International Journal for Quality in Health Care. 2011; mzr013. 10.1093/intqhc/mzr013 21467077

[15] United Nations Development Program. Assessing Progress toward the Millennium Development Goals: Ethiopia MDG Report 2012. 2012.

[16] Curry LA, Byam P, Linnander E, Andersson KM, Abebe Y, Zerihun A, et al Evaluation of the Ethiopian Millennium Rural Initiative: Impact on Mortality and Cost-Effectiveness. 2013. 10.1371/journal.pone.0079847 PMC3832618 24260307

